# Transition into adult care: factors associated with level of preparedness among adolescents living with HIV in Cambodia

**DOI:** 10.1186/s12981-017-0159-6

**Published:** 2017-07-17

**Authors:** Siyan Yi, Chanrith Ngin, Khuondyla Pal, Vohith Khol, Sovannary Tuot, Sokunmealiny Sau, Pheak Chhoun, Gitau Mburu, Sok Chamreun Choub, Kolab Chhim, Penhsun Ly

**Affiliations:** 1 KHANA Center for Population Health Research, No. 33, Street 71, Phnom Penh, Cambodia; 2 Center for Global Health Research, Touro University California, Vallejo, USA; 3 National Center for HIV/AIDS, Dermatology and STD, Phnom Penh, Cambodia; 4 Division of Health Research, Lancaster University, Lancaster, UK

**Keywords:** Adolescents, HIV, AIDS, Care and treatment, Transition, Cambodia

## Abstract

**Background:**

Preparing adolescents for transition into adult care and supporting their acquisition of self-health care management skills is a critical determinant of their post-transition HIV care outcomes. However, there is a scarcity of research on effective transition strategies. This study explores factors associated with adolescent preparedness for transition into adult care in Cambodia.

**Methods:**

In August 2016, a cross-sectional study was conducted among 223 adolescents living with HIV aged 15–17, randomly selected from 11 antiretroviral therapy clinics, utilizing a structured questionnaire. The level of preparedness was determined using a pre-existing scale, and adolescents were categorized as having a high- or low level of preparedness for transition. Bivariate and multivariate analyses were conducted.

**Results:**

Of 223 adolescents, 55.2% were male, and their mean age was 15.8 years. Overall, 53.3% had a high level of preparedness for transition. As part of the transition protocol, 2.7% had completed a transfer form, 24.7% had a transition case manager, 29.6% had been counselled about the transition, and 19.7% had visited an adult ART clinic. In multivariate analysis, a higher level of preparedness for transition was independently associated with older age (AOR 2.44, 95% CI 1.34–4.46; *p* = 0.004), family having received social support for their health (AOR 5.32, 95% CI 1.97–14.36; *p* = 0.001), knowing the kind of treatment they received (ART) (AOR 12.67, 95% CI 2.91–15.19; *p* = 0.001), trust in friends or family for HIV treatment (AOR 7.82, 95% CI 1.13–8.89; *p* = 0.008), receiving counseling on transition (AOR 3.17, 95% CI 1.15–8.76; *p* = 0.03), having a ‘Case Manager’ identified to support them during the preparation process for transition (AOR 3.89, 95% CI 1.08–13.96; *p* = 0.04), and satisfaction with preparation process for transition in general (AOR 0.35, 95% CI 0.03–0.87; *p* = 0.01).

**Conclusions:**

A range of individual, social and health system and services factors may determine successful transition preparedness among adolescents in Cambodia. Strengthening implementation of age-appropriate and individualized case management transition at all sites, while creating supportive family, peer, and healthcare environments for adolescent transition is required.

## Background

Globally, adolescents and young people represent a growing share of people living with human immunodeficiency virus (HIV). Latest available data suggests that adolescents account for 12% of new HIV infections globally [1]. Approximately 670,000 young people between the ages of 15–24 were newly infected with HIV in 2015 alone, of whom 250,000 were adolescents between the ages of 15 and 19 [1]. About 1.8 million adolescents between the ages of 10 and 19 were living with HIV worldwide in 2015 [1], and this number is projected to increase further due to rising access to antiretroviral therapy (ART) and ongoing new horizontal infections [2, 3]. The Pacific and South East Asia regions have the highest number of adolescents living with HIV outside of Sub-Saharan Africa, accounting for 11% of the global population of adolescents living with HIV [4]. As in other contexts [3, 5], majority of these adolescents have been vertically infected [4].

Although data on adolescents living with HIV are limited globally [6], concerns have emerged regarding their HIV-related clinical outcomes. While the overall mortality among people living with HIV in all other age groups had decreased by 32% during the period between 2005 and 2012, the overall mortality of adolescents living with HIV increased by 50% from 71,000 in 2005 to 110,000 in 2012 [6]. Adolescents are the only age group in which HIV-related mortality is rising [7].

Compared to adults, adolescents often have poorer rates of viral suppression [8, 9] and long-term immunologic recovery [10]. They face unique developmental, psychological, and sexuality challenges as they are still maturing physically, mentally, and sexually [5, 11], often in the context of restrictive parental consent laws and policies [12]. This is in addition to common contextual challenges encountered by most people living with HIV regardless of age, including stigma [13], disclosure difficulties [13], challenges with adherence [14], and economic hardships [15], all of which prevent attainment of optimal health outcomes. As a consequence, adolescents are considerably less likely to receive HIV services compared to adults. In 2013, less than one in four (24%) children up to 15 years living with HIV had received ART globally [16].

Recent research has identified transition to adult care as a significant determinant of HIV care outcomes among adolescents living with HIV [17]. For instance, studies have demonstrated that transition to adult HIV care is associated with worsening or reversal of immunological recovery [18], unsuppressed viral load [19, 20], and high rates of attrition and loss to follow up [20, 21] among transitioned adolescents. These concerns have led to calls for programs and policies to improve adolescent transition processes, data and outcomes [17, 22].

According to the World Health Organization (WHO), transition preparedness is determined by adolescents’ ability to demonstrate basic health-care awareness, awareness of their status, understanding their disease, and development of own health management skills [23]. Few data exist on what approaches are most effective for preparing and implementing transition [17]. Meanwhile, there is consensus that adolescents cannot self-manage a chronic illness when they do not understand its nature and clinical management [24], and that there is a need for programs for adolescents that are empowering, continuous, coordinated, culturally appropriate, integrated and family-centered [17, 25].

Recent studies have also revealed a lack of consensus and consistency regarding specific elements of an ideal transition program [26]. Even in the contexts where transition is being implemented, there has been a pervasive lack of mechanisms to assess the impact of different transition approaches [26, 27]. Studies involving adolescents and health care providers have also demonstrated differing perceptions of readiness for transition [28] amid calls to assess adolescent readiness for transition [29]. These difficulties are not surprising. The issue of transition has unexpectedly been brought to fore due to increasing survival of perinatally-infected children, against a prior assumption that they would not survive into adulthood [30]. Although studies looking at survival of adolescents after transition have emerged prompted by the rising numbers of this population, most focus on experiences, retention and retrospective clinical outcomes in developed countries [27, 31–34], leaving a gap of information related to transition processes in low and middle income countries.

In Cambodia, where HIV prevalence in pregnant women attending antenatal care was 0.28% in 2015 [35], there were 8512 children and adolescents living with HIV at the end of 2013 [36], who accessed care from 37 ART clinics across the country. A previous exploratory study conducted in 2012 suggested that the majority of these adolescents were reluctant to transition into adult services [37] due to anticipated stigma, lack of familiarity with adult clinics, current relationship with pediatric health providers, negative perception of adult service quality, and concerns regarding confidentiality [37, 38]. These factors, combined with inability of adult health care providers to receive adolescents revealed in the above study formed the basis for calls to increase attention to adolescent transition in Cambodia, including better data analyses and operational research around transition [39].

To address these needs and imperatives, a study was conducted in 2016 to explore potential factors associated with level of preparedness for transition to adult care among adolescents living with HIV in Cambodia.

## Methods

### Study design and population

This overall study constituted of a cross-sectional survey and follow on in-depth interviews complemented by routine HIV care data retrieved from medical records in ART clinics. This study included adolescents living with HIV aged 15–17, who were accessing services from pediatric ART clinics in the capital city and 12 provinces. In this paper, we report the findings from the quantitative arm. Qualitative results have been reported separately.

### Selection of study sites and participants

In Cambodia, 90% of adolescents living with HIV access services from 18 clinics, while the remaining 10% are distributed across 21 sites, each with less than 10 adolescents living HIV clients. At the time of the study, the number of adolescents per ART clinic varies from 1 to 116. Against this background, participants were selected in three stages. First, a two-stage 30 × 10 cluster sampling method was used to select clusters to sample participants from, using a methodology developed by the WHO [40]. A comprehensive list of all ART sites in the country with adolescents living with HIV population estimates was created and used to select the clusters after excluding ART clinics with less than 10 adolescents. This left a universe of 598 adolescents receiving treatment and care from the 18 major ART clinics. To minimize costs of data collection, additional seven clinics with less than 20 adolescents living with HIV clients aged 15–17 were excluded.

In the second stage the required sample size was determined taking into account population size, confidence interval, and estimated proportion of the population with the characteristics of interest [41], in this case being prepared for transition to adult care. Assuming 60% of adolescents had a high level of preparedness, a finite population size of 598, a design effect of 1.4 and a 10% drop-out or refusal rate, a minimum sample size of 220 was required for the study to measure variables among selected adolescents with a confidence interval of 4.3%.

Third, the number of participants that needed to be recruited from each cluster was determined. Because the size of the ART clinics varied, probability proportional to size sampling method [41] was used to ensure that adolescents in larger sites had the same probability of being included in the sample as those in smaller sites. Cumulative population size of each ART clinic was calculated; sampling interval was determined; and potential participants were randomly selected from the database using a random number table.

Finally, a list of selected adolescents was prepared and assigned a unique identification number based on the clinic ID code. Selected adolescents were then contacted on phone, screened for eligibility, and informed about the research objectives as well as place, date, and time of the interview by local study coordinators. A written consent was obtained from parents or guardians of selected adolescents before the interview. Potential participants were included if they were aged between 15 and 17 years, receiving treatment and care services from the selected ART clinics, able to communicate in Khmer, allowed by a parent or guardian to participate, able to present themselves on the day of the interview, and physically and mentally stable to assent and participate in the study. Recruitment continued until the required sample sizes for each cluster and clinics were achieved.

### Questionnaire development

In reference to exiting literature [28, 42], a structured questionnaire was developed. The first part of the questionnaire collected information on socio-demographic characteristics, parental or caregiver information, and clinical and immunological data of participants obtained from health records. The second part collected information on adolescents’ HIV knowledge, substance use and sexual behaviors. The third part assessed the level of preparedness for transition to adult care, using a questionnaire developed by the New York State Department of Health AIDS Institute [43]. This 11-item scale asked whether the adolescents: (1) could recognize when they are getting sick, (2) knew when they need to call the doctor, (3) were responsible for making appointments, (4) were responsible for refilling medications, (5) felt comfortable asking questions at appointments, (6) had a copy of your health records, doctor contact number, and address, (7) had a method of keeping track of healthcare appointments, (8) found it difficult to remember to take medicines, (9) would stop taking your medicine when they felt better, (10) would stop taking if they felt worse after taking medicines, and (11) had missed taking any medicines in the past 4 days. A total score of the scale, which ranged from 0 to 11, was calculated for each participant, with a Cronbach’s alpha of 0.86. The final part of the questionnaire collected information on coping strategies and the maturity of the adolescents.

### Data collection training

Data were collected by two teams of interviewers and moderators who were trained on the study protocol, research ethics, confidentiality and interview and questionnaire administration skills. Tool pre-testing was conducted among 20 adolescents living with HIV recruited from an ART clinic in Phnom Penh and 10 parents/guardians, followed by revision of contents and language based on feedbacks from the pre-test.

### Data analyses

Data were coded and scaled continuously or categorically as dictated by the variables and entered into a computerized database using Epi Data version 3 (Odense, Denmark). Double data entry was performed to minimize errors. Descriptive analyses were conducted to determine proportions of variables, frequency distribution of responses, and means and standard deviation (SD) of continuous variables.

Because there was no recommended cut-off for determining level of preparedness for transition to adult care in the scale employed in this study [43], the mean score was used to categorize participants into two groups: one with a low level of readiness (with a total score of ≤7) and another group with a high level of readiness (with a total score of >7). Chi square test, Fisher’s exact test, or *t* test were used as appropriate based on the nature of variables being analyzed to compare variables among adolescents with a low and high level of preparedness for transition to adult care. *p* values of less than 0.05 were considered significant.

A multivariate logistic regression model was constructed to identify factors associated with a high level of preparedness for transition. Age, gender, and all other variables associated with a high level of readiness in bivariate analyses at a level of *p* value <0.05 were simultaneously included in the model, followed by a backward selection to identify independent predictors. SPSS v22 (IBM Corporation, New York, USA) was used for all statistical analyses.

### Ethics statement

This study was approved by the National Ethics Committee for Health Research, Ministry of Health in Cambodia (Ref: 297NECHR). All participants were required to assent to the study in writing and a written consent from their parent(s) or caregiver giver was obtained. All data were collected in private locations, and confidentiality strictly protected by removing all personal identifiers from the questionnaires, field notes, and the sampling frame. Participants were assigned a unique code. Researchers and data collectors were trained on confidentiality prior to data collection. All participants were provided with a token of 2.5–5 USD for transportation.

## Results

### Socio-demographic characteristics

Socio-demographic characteristics are shown in Table 1. A total of 223 adolescents were included in this study. Of these 55.2% were male, and their mean age was 15.8 years (SD = 0.8). 29.6% had completed primary school, and 50.7% had completed secondary school. Although 47.5 and 42.6% reported having a living mother and father, respectively, only 40.8% were living with parents, and the remaining were living with grandparents (15.7%) and with other relatives (33.6%). Almost 10% lived in an orphanage, with Buddhist monks, neighbors, or foster parents. Main caregivers included parents (55.6%), relatives (32.2%), siblings (6.7%), orphanage/NGO staff (3.3%), and grandparents (2.2%). About one-fifth (22.0%) were working for pay, and 48.4% came from families that received social support for their health care, in the form of food support (78.7%), school allowance (64.8%), transport allowance for going to ART clinic (55.6%), emotional counseling (32.4%), vocational training (25.9%), or home visit (10.2%).

**Table 1:** Socio-demographic characteristics of adolescents living with HIV with a low and high level of preparedness for transition to adult care

Socio-demographic characteristics	Total (*n* = 223)	Level of preparedness for transition^a^		
		Low (≤7) (*n* = 104)	High (>7) (*n* = 119)	*p* value^†^
	*n* (%)	*n* (%)	*n* (%)	
Age (in years)	15.8 ± 0.8	15.7 ± 0.8	15.9 ± 0.8	0.16
Gender				0.09
Male	123 (55.2)	51 (49.0)	72 (60.5)	
Female	100 (44.8)	53 (51.0)	47 (39.5)	
Currently living with				0.004
Parents	91 (40.8)	30 (28.8)	61 (51.3)	
Grandparents	35 (15.7)	18 (17.3)	17 (14.3)	
Relatives	75 (33.6)	47 (45.2)	28 (23.5)	
In an orphanage	18 (8.1)	7 (6.7)	11 (9.2)	
Other	4 (1.8)	2 (1.9)	2 (1.7)	
Mother is still alive				0.04
No	117 (52.5)	62 (59.6)	55 (46.2)	
Yes	106 (47.5)	42 (40.4)	64 (53.8)	
Mother’s level of formal education				0.59
No education	12 (11.7)	5 (12.5)	7 (11.1)	
Primary school	26 (25.2)	9 (22.5)	17 (27.0)	
Secondary school	14 (13.6)	8 (20.0)	6 (9.5)	
High school or higher	20 (19.4)	8 (20.0)	12 (19.0)	
Don’t know	31 (30.1)	10 (25.0)	21 (33.3)	
Father is still alive				0.37
No	128 (57.4)	63 (60.6)	65 (54.6)	
Yes	95 (42.6)	41 (39.4)	54 (45.4)	
Father’s level of formal education				0.62
No education	4 (4.4)	1 (2.6)	3 (5.6)	
Primary school	21 (23.1)	10 (25.6)	11 (21.2)	
Secondary school	11 (12.1)	4 (10.3)	7 (13.5)	
High school or higher	19 (20.9)	20 (15.4)	13 (25.0)	
Don’t know	36 (39.6)	18 (46.2)	18 (34.6)	
Main daily caregiver				0.003
Parent	124 (55.6)	48 (46.2)	76 (63.9)	
Grand parent	5 (2.2)	2 (1.9)	3 (2.5)	
Sibling	15 (6.7)	4 (3.8)	11 (9.2)	
Relatives	72 (32.3)	47 (45.2)	25 (21.0)	
Orphanage/NGO staff	7 (3.1)	3 (2.9)	4 (3.4)	
Main caregiver’s level of formal education				0.08
Primary school or lower	7 (5.0)	6 (7.9)	1 (1.6)	
Secondary school	29 (20.9)	13 (17.1)	16 (25.4)	
High school	9 (6.5)	7 (9.2)	2 (3.2)	
University or higher	21 (15.1)	14 (18.4)	7 (11.1)	
Don’t know	73 (52.5)	36 (47.4)	37 (58.7)	
Your level of formal education				0.49
Primary school or lower	66 (29.6)	32 (30.8)	34 (28.6)	
Secondary school	113 (50.7)	55 (52.9)	58 (48.7)	
High school or higher	44 (19.7)	17 (16.3)	27 (22.7)	
Currently working for pay				0.03
No	174 (78.0)	88 (84.6)	86 (72.3)	
Yes	49 (22.0)	16 (15.4)	33 (27.7)	
Family received social support for your health				0.03
No	115 (51.6)	62 (59.6)	53 (44.5)	
Yes	108 (48.4)	42 (40.4)	66 (55.5)	
Types of social support				
Transportation allowance	60 (55.6)	26 (61.9)	34 (51.5)	0.29
Food support	85 (78.7)	37 (88.1)	48 (72.7)	0.06
School allowance	70 (64.8)	28 (66.7)	42 (63.6)	0.75
Emotional counseling	35 (32.4)	21 (50.0)	14 (21.2)	0.002
Vocational training	28 (25.9)	18 (42.9)	10 (15.2)	0.001
Home visit	11 (10.2)	3 (7.1)	8 (12.1)	0.52

Values are number (%) for categorical variables and mean (±SD) for continuous variables

*ART* antiretroviral therapy, *HIV* human immunodeficiency virus, *NGO* non-governmental organization

^†^Chi square or Fisher’s exact test was used for categorical outcome variables and Student’s *t* test was used for continuous outcome variables

^a^Mean score of preparedness for transition scale was used to divide participants into two groups—adolescents with low level of readiness (≤7) and adolescents with high level of readiness (>7)

### Immunological and adherence characteristics

As shown in Table 2, based on their medical records obtained at the ART clinic, participants had received services from an ART clinic for an average of 114 (SD = 39) months, and had received ART for 101 (SD = 40) months. The mean initial CD4 count was 632 (SD = 506) cells/mm^3^, and the most recent was 672 (SD = 295) cells/mm^3^. The mean viral load at the first test was 44,691 (SD = 161,304) copies/ml, and at the most recent was 7686 copies/ml (SD = 48,682) copies/ml.

**Table 2:** Medical history among adolescents living with HIV with a low and high level of preparedness for transition to adult care

Medical history	Total (*n* = 223)	Level of preparedness for transition^a^		
		Low (≤7) (*n* = 104)	High (>7) (*n* = 119)	*p* value^†^
	*n* (%)	*n* (%)	*n* (%)	
Type of ART site				
Pediatric	206 (92.8)	67 (95.7)	139 (92.1)	0.59
Adult	10 (4.5)	2 (2.86)	7 (4.6)	
Other	6 (2.7)	1 (1.4)	5 (3.3)	
Type of current treatment				0.87
First line	183 (82.4)	58 (84.1)	124 (81.6)	
Second line	36 (16.2)	11 (15.9)	25 (16.4)	
Opportunistic infections	3 (1.4)	0 (0.0)	3 (2.0)	
Time since first ART clinic visit (in months)	114 ± 39	111 ± 38	116 ± 39	0.33
Time since first ART initiation (in months)	101 ± 40	100 ± 41	102 ± 41	0.66
Time from first visit to ART start (in months)	14 ± 25	12 ± 22	15 ± 26	0.50
First CD4 count	632 ± 506	623 ± 523	636 ± 502.4	0.86
Latest CD4 count	672 ± 295	717 ± 309	655 ± 286	0.15
First viral load count (copies)	44,691 ± 161,304	38,015 ± 139,375	47,922 ± 171,630	0.68
Latest viral load count (copies)	7686 ± 48,682	10,306 ± 77,534	6547 ± 27,418	0.61
Visual adherence scale (%)	95.6 ± 9.8	93.8 ± 10.7	97.1 ± 8.6	0.01

Values are number (%) for categorical variables and mean (± SD) for continuous variables

*ART* antiretroviral therapy, *HIV* human immunodeficiency virus

^†^Chi square or Fisher’s exact test was used for categorical outcome variables and Student’s *t* test was used for continuous outcome variables

^a^Mean score of preparedness for transition scale was used to divide participants into two groups—adolescents with low level of readiness (≤7) and adolescents with high level of readiness (>7)

### Awareness and disclosure of HIV status

Majority (93.7%) of the participants in this study were aware that they were living with HIV, and 78.9% reported that they had acquired it from their mothers. Half (50.7%) had never disclosed their HIV status to anyone, and the remaining 49.3% had disclosed it to their siblings (24.2%), friends (13.0%), school teachers (2.4%), or other persons (5.8%). Based on self-reported visual adherence scale, 95.6% were adherent to ART (Table 3).

**Table 3:** Assessment of HIV status disclosure among adolescents living with HIV with a low and high level of preparedness for transition to adult care

HIV status disclosure	Total (*n* = 223)	Level of preparedness for transition^a^		
		Low (≤7) (*n* = 104)	High (>7) (*n* = 119)	*p* value^†^
	*n* (%)	*n* (%)	*n* (%)	
Can you tell us, what is your disease?				0.17
HIV/AIDS	209 (93.7)	95 (91.3)	114 (95.8)	
Don’t know	14 (6.3)	9 (8.7)	5 (4.2)	
Do you know how you have been infected?				0.78
Don’t know	31 (14.8)	13 (13.7)	18 (15.8)	
Mother to child	165 (78.9)	77 (81.1)	88 (77.2)	
Other	13 (6.2)	5 (5.3)	8 (7.0)	
Do you know how is this disease transmitted?				
Don’t know	7 (3.3)	7 (7.4)	0 (0.0)	0.004
Mother to child	142 (67.9)	65 (68.4)	77 (67.5)	0.89
Unprotected sex	156 (74.6)	66 (69.5)	90 (78.9)	0.12
Sharing needles	114 (54.5)	50 (52.6)	64 (56.1)	0.61
Blood transfusion	125 (59.8)	64 (57.4)	61 (53.5)	0.05
Do you know what kind of medicine you have received?				0.002
Pre-ART	3 (1.3)	1 (1.0)	2 (1.7)	
ART	183 (82.1)	76 (73.1)	107 (89.9)	
Don’t know	37 (16.6)	27 (26.0)	10 (8.4)	
Have you ever disclosed your HIV status to anyone?				0.04
No	113 (50.7)	60 (67.7)	53 (44.5)	
Yes	110 (49.3)	44 (42.3)	66 (55.5)	
To whom have you ever disclosed your HIV status to?				0.06
Siblings	50 (24.2)	14 (14.9)	36 (31.9)	
School teachers	5 (2.4)	2 (2.1)	3 (2.7)	
Friends	27 (13.0)	12 (12.8)	15 (13.3)	
Other	12 (5.8)	6 (6.4)	6 (5.3)	

*AIDS* acquired immune deficiency syndrome, *ARV* antiretroviral, *HIV* human immunodeficiency virus

^†^Chi square or Fisher’s exact test was used as appropriate

^a^Mean score of preparedness for transition scale was used to divide participants into two groups

### HIV and health information sources

Table 4 shows that 60.1% of the adolescent participants reported ART clinic to be the main source of health information. Family (45.3%), and health providers (22.4%), were preferred by most adolescents to discuss issues of health, sexual life, or daily life, compared to friends (16.6%), counselors/peer educators (4.0%), or caregivers, relatives, and school teachers (11.7%). However, health care providers (78.9%) were most trusted in regard to HIV treatment and care, compared to family (12.1%), counselors/peer educators (4.5%), caregivers and relatives (4.5%).

**Table 4:** Experience of preparation for transition among adolescents living with HIV with a low and high level of preparedness for transition to adult care

Experiences in preparation process for transition	Total (*n* = 223)	Level of preparedness for transition^a^		
		Low (≤7) (*n* = 104)	High (>7) (*n* = 119)	*p* value^†^
	*n* (%)	*n* (%)	*n* (%)	
Facility you prefer to receive HIV treatment and care				0.08
Pediatric ART services	187 (83.9)	92 (88.5)	95 (79.8)	
Adult ART services	36 (16.1)	12 (11.5)	24 (20.2)	
Preferred to discuss questions related to health, sexual life, or daily life with				0.78
Health care providers	50 (22.4)	21 (20.2)	29 (24.4)	
Counselors/peer educators	9 (4.0)	5 (4.8)	4 (3.4)	
Friends	37 (16.6)	20 (19.2)	17 (14.3)	
Family	101 (45.3)	45 (43.3)	56 (47.1)	
Other	26 (11.7)	13 (12.5)	13 (10.9)	
Person who you trust the most for your treatment				0.001
Health care providers	176 (78.9)	92 (88.5)	84 (70.6)	
Counselors/peer educators	10 (4.5)	5 (4.8)	5 (4.2)	
Friends/family	27 (12.1)	3 (2.9)	24 (20.2)	
Other	10 (4.5)	4 (3.8)	6 (5.0)	
Sources of information about health				0.90
ART clinic	134 (60.1)	61 (58.7)	73 (61.3)	
NGOs	17 (7.6)	8 (7.7)	9 (7.6)	
Family	51 (22.9)	26 (25.0)	25 (21.0)	
Other	21 (9.4)	9 (8.7)	12 (10.1)	
Received counseling on transition to adult services				0.01
No	157 (70.4)	82 (78.8)	75 (63.0)	
Yes	66 (29.6)	22 (21.2)	44 (37.0)	
Person who provided the counseling				0.43
Health providers	24 (36.9)	7 (33.3)	17 (38.6)	
Counselors/peer educators	31 (47.7)	9 (42.9)	22 (50.0)	
Other	10 (15.4)	5 (23.8)	5 (11.4)	
Ever completed a transfer form				0.14
No	217 (97.3)	103 (99.0)	114 (95.8)	
Yes	6 (2.7)	1 (1.0)	5 (4.2)	
Ever visited an adult clinic to prepare for transition				0.03
No	179 (80.3)	90 (86.5)	89 (74.8)	
Yes	44 (19.7)	14 (13.5)	30 (25.2)	
Person who took you to visit the adult clinic to prepare for transition				0.15
Counselors/peer educators	2 (4.7)	1 (7.7)	1 (3.4)	
Friends/family	30 (69.8)	11 (84.6)	19 (63.3)	
Other	11 (25.5)	1 (7.7)	10 (33.3)	
The visit was helpful for you to cope with the transition				0.61
No	5 (11.6)	2 (15.4)	3 (10.0)	
Yes	38 (88.4)	11 (84.6)	27 (90.0)	
A ‘Case Manager’ has been identified to support you during the transition				<0.001
No	168 (75.3)	91 (87.5)	77 (64.7)	
Yes	55 (24.7)	13 (12.5)	42 (35.3)	
Preparedness to manage your treatment going forward				0.01
Very prepared	29 (13.0)	8 (7.7)	21 (17.6)	
Somewhat prepared	168 (75.3)	81 (77.9)	87 (73.1)	
Somewhat unprepared	12 (5.4)	4 (3.8)	8 (6.7)	
Very unprepared	14 (6.3)	11 (10.6)	3 (2.5)	
Feeling supported during preparation process for the transition to adult care				0.96
Very supported	11 (14.9)	2 (11.8)	9 (15.8)	
Somewhat supported	44 (59.5)	10 (58.8)	34 (59.6)	
Somewhat unsupported	7 (9.4)	2 (11.8)	5 (8.8)	
Very unsupported	12 (16.2)	3 (17.6)	9 (15.8)	
Satisfaction you with the preparation process for transition in general				<0.001
Very satisfied	16 (7.2)	2 (1.9)	14 (11.8)	
Somewhat satisfied	51 (22.9)	15 (14.4)	36 (30.3)	
Somewhat dissatisfied	26 (11.7)	11 (10.6)	15 (12.6)	
Very dissatisfied	130 (58.3)	76 (73.1)	54 (45.4)	

*ART* antiretroviral therapy, *HIV* human immunodeficiency virus

^†^Chi square or Fisher’s exact test was used as appropriate

^a^Mean score of preparedness for transition scale was used to divide participants into two groups—adolescents with low level of readiness (≤7) and adolescents with high level of readiness (>7)

### Preparation process for transition into adult care

Table 4 shows that a total of 29.6% had received counseling on transition into adult services, of which majority were counselled by health care providers (36.9%) and counselors/peer educators (47.7%). However, only 19.7% had visited an adult ART clinic to prepare for the transition with friends/family (69.8%), counselors/peer educators (4.7%), or other including NGO staff (25.5%). In addition, only 2.7% of them had completed a transfer form. Most of the adolescents who had visited the adult facility (88.4%) reported that the visit was helpful in preparing for the transition. In addition, 24.7% reported that a ‘Case Manager’ had been identified to support them for the transition. When asked about their preparedness to manage their treatment going onward, 13.0% said they were very prepared; 75.3% were somewhat prepared; 5.4% were somewhat unprepared; and 6.3% were very unprepared. The majority felt supported during the preparation process for transition with 14.5% feeling very supported, and 59.5% felt somewhat supported. However, 58.3% said that they were very dissatisfied, and 11.7% said that they were somewhat dissatisfied with the preparation process in general. Despite these findings, the majority (83.9%) of participants preferred to receive HIV treatment and care in a pediatric ART clinic.

### Assessment of adolescents’ preparedness for transition into adult care

**Fig. 1:**
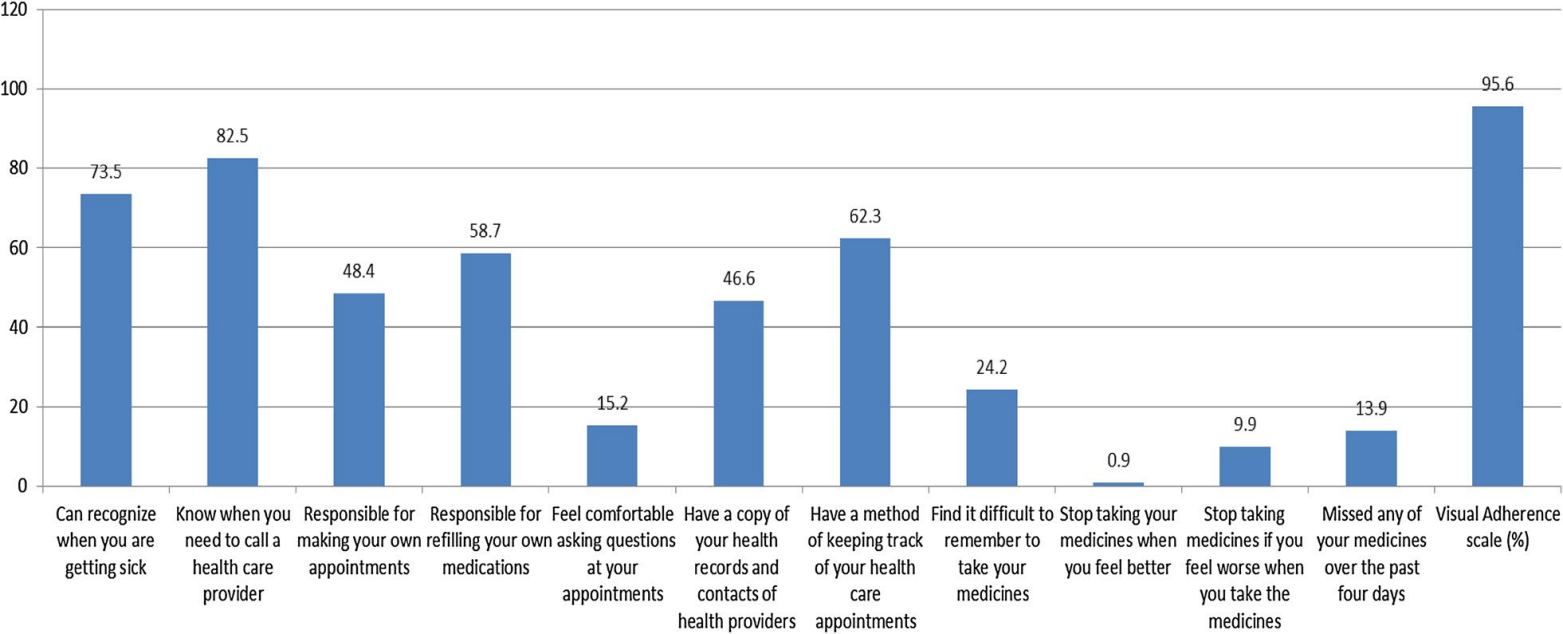
Assessment of preparedness for the transition from pediatric to adult care among adolescents living with HIV

Based on the scale employed in this study [43], 53.3% had a high level of preparedness for transition to adult care. As shown in Fig. 1, majority of the participants reported that they could recognize when they are getting sick (73.5%), knew when they needed to call a health care provider (82.5%), and had a method of keeping track of their clinic appointments (62.3%). However, smaller proportions reported being responsible for making their actual clinic appointments (48.4%), refilling their own medications (58.7%), or feeling comfortable asking questions during clinic appointments (15.2%). Less than half (46.6%) reported having a copy of their health records and contacts of health care providers. Regarding ART adherence, 24.2% found it difficult to remember to take medicines, but only a small proportion would stop taking medicines if they feel better (0.9%) or worse (9.9%). A total of 13.9% reported either having missed or being uncertain of whether they had missed their medicines over the past 4 days preceding the interview, with a mean score of visual adherence scale of 95.6% (SD = 9.8%).

### Factors associated with level of preparedness for transition into adult care

Bivariate analyses showed that adolescents with a higher level of preparedness were significantly more likely to live with their parents (28.8% vs. 51.3%, *p* = 0.004), to have a living mother (40.4% vs. 53.8%, *p* = 0.04), and to have their parent as their main caregiver (46.2% vs. 63.9%, *p* = 0.003), while adolescents with a lower level of preparedness were more likely to live with relatives (45.2% vs. 21.0%, *p* = 0.003) (Table 1). In addition, the level of adherence was significantly higher among adolescents with a higher level of preparedness on bivariate analysis (93.8 ± 10.7 vs. 97.1 ± 8.6, *p* = 0.01) (Table 2). Adolescents with a lower level of preparedness were significantly more likely to report that they did not know how their disease was transmitted (7.4% vs. 0.0%, *p* = 0.004), to not know the kind of medicines they were receiving (20.6% vs. 8.4%, *p* = 0.002), and to have disclosed their HIV status to someone (42.3% vs. 55.5%, *p* = 0.04). Conversely, adolescents with a higher level of preparedness knew that they were receiving ART (73.1% vs. 89.9%, *p* = 0.002) (Table 3).

Adolescents with a lower level of preparedness were significantly more likely to trust health care providers with their HIV treatment and care (88.5% vs. 70.6%, *p* = 0.001), while adolescents with a higher level of preparedness identified friends or family in this regard (2.9% vs. 20.2%, *p* = 0.001). A higher level of preparedness was associated with receiving counseling on transition into adult services (21.2% vs. 37.0%, *p* = 0.01) and having visited an adult clinic in preparation for transition (13.5% vs. 25.2%, *p* = 0.03). Presence of a ‘Case Manager’ to support adolescents during transition was also associated with a higher level of preparedness (12.5% vs. 35.3%, *p* < 0.001). Not surprisingly, adolescents with a higher level of preparedness perceived that they were very prepared for the transition (7.7% vs. 17.6%, *p* = 0.01), while those with a lower level of preparedness reported that they were very dissatisfied with the preparation process (73.1% vs. 45.4%, *p* < 0.001) (Table 4).

As shown in Table 5, multivariate logistic regression analysis found that a higher level of preparedness remained significantly associated with older age (AOR 2.44, 95% CI 1.34–4.46), family having received social support for their health (AOR 5.32, 95% CI 1.97–14.36), knowing the kind of treatment received (ART) (AOR 12.67, 95% CI 2.91–15.19), trusting friends or family for HIV treatment (AOR 7.82, 95% CI 1.13–8.89), receiving counseling on transition to adult services (AOR 3.17, 95% CI 1.15–8.76), having a ‘Case Manager’ to support them for the transition (AOR 3.89, 95% CI 1.08–13.96), and satisfaction with preparation process for transition in general (AOR 0.35, 95% CI 0.03–0.87).

**Table 5:** Factors associated with level of preparedness for transition to adult care in multivariate logistic regression model

Variables in the final model^a^	Level of preparedness for transition^b^	
	AOR (95% CI)	*p* value
Age	2.44 (1.34–4.46)	0.004
Family received social support for your health		
No	Reference	
Yes	5.32 (1.97–14.36)	0.001
Do you know what kind of medicine you have received?		
Don’t know	Reference	
Pre-ART	4.20 (1.99–7.39)	0.02
ART	12.67 (2.91–15.19)	0.001
Person who you trust the most for your treatment		
Health providers	Reference	
Counselors/peer educators	2.41 (0.42–13.79)	0.33
Friends/family	7.82 (1.13–8.89)	0.008
Other	1.51 (0.23–9.78)	0.66
Received counseling on transition to adult services		
No	Reference	
Yes	3.17 (1.15–8.76)	0.03
A ‘Case Manager’ has been identified to support you during the transition		
No	Reference	
Yes	3.89 (1.08–13.96)	0.04
Satisfaction with preparation for transition in general		
Very satisfied	Reference	
Somewhat satisfied	0.21 (0.08–5.46)	0.35
Somewhat dissatisfied	0.32 (0.01–0.94)	0.04
Very dissatisfied	0.35 (0.03–0.87)	0.01

*AOR* adjusted odds ratio, *ART* antiretroviral therapy, *CI* confidence interval

^a^Age, gender and all variables associated with level of preparedness for transition to adult care at a level of p < 0.05 were simultaneously included in a multivariate logistic regression model

^b^Mean score of readiness for transition scale was used to divide participants into two groups—adolescents with low level of readiness (≤7) and adolescents with high level of readiness (>7)

## Discussion

Preparing adolescents for transition into adult care and supporting their acquisition of self-health care management skills is an essential part of transition protocols [44, 45], which affects outcomes after transition [31, 34, 45, 46]. This is the first study exploring factors associated with preparedness for transition from pediatric to adult care among adolescents living with HIV in Cambodia. A number of findings that merit program and policy attention.

First, the overall level of preparedness for transition from pediatric to adult care among adolescents living with HIV in this study was fairly good. The results indicated that although few were undertaking practical activities associated with taking control of their HIV management such as booking their own appointment, refilling their own medications, keeping a copy of their health cards, a good number of them felt confident that they could recognize when they are getting sick and know when they need to call a health care provider. It is, however, difficult to know for certain if adolescents will be comfortable with these practical tasks in the wake of transition. A good transition process enables adolescents to be autonomous and responsible for their own HIV care by the time they engage with adult services [46], and as such ongoing support is required until and after they start accessing adult care, and afterwards.

Second, this study provides important information regarding individual, social and health system determinants of preparedness to transition, which should be considered moving forward. As other researchers have asserted, the preparation for transition ought to take into account the characteristics and contexts of adolescents and their families; physical, sexual, and emotional maturity; complexity of their health needs; and access to health care professionals and services [25, 45, 49]. In a concentrated epidemic context such as Cambodia, being an adolescent from a key population can exacerbate stigma and other barriers to successful transition [20].

At the individual domain, a higher level of preparedness for transition into adult care was independently associated with older age and being aware of the kind of treatment received. While the prominence of age is not necessarily surprising, having been highlighted in other studies [34, 47], these findings do emphasize that tailored, age- and developmental appropriate transition should recognize the evolving capacity of adolescents [45]. This may have a gender-implication given that female adolescents, who generally mature earlier than their male counterparts were older in this study. In addition, this finding emphasizes the need to coordinate the processes of disclosure and transition, ensuring that adolescents are fully aware of the nature of disease and treatment before transition, which will require focussed and differentiated HIV and treatment literacy interventions for adolescents as they approach transition into adult care.

At the social level, a higher level of preparedness for transition into adult care was independently associated with trust in friends or family for HIV treatment, and receipt of health-related social support at the family level such as emotional counseling and vocational training. These findings are consistent with other studies showing the importance of supportive family members, peers, and friends and a communicative family environment in coping with both an HIV diagnosis [49] and transition to adult care [50]. These findings are particularly important given the high level of stigma [38], and the large proportion of adolescents not living with their parents in the study context, implying the necessity for interpersonal support.

In relation to health systems and services, receiving specific counseling on transition to adult services, having a Case Manager to support adolescents for transition preparation was associated with higher levels of preparedness. Other studies have consistently shown the importance of dedicated health and social workers to support transition in the United States [51–53], and having trusting relationships with health providers [25, 30], which may often require additional training to improve competency of health providers [30] to enable them to receive adolescents and create atmosphere which enables communication and questions from adolescents. Creating familiarity with adult clinics, including through visits has a significant impact in facilitating adolescents’ connection to the adult clinic consistent with research from other settings [33]. In our study, 88% of participants who had visited an adult clinic as part of the preparation for transition reported that they found the visit helpful, although only a fifth of the study sample had been to the clinic. In addition, despite the importance of transition case management, less than a quarter (24.7%) of the participants had an identified Case Manager, and only a third (29.6%) had been counselled about the transition process.

Third, these results emphasize calls to take into account adolescents views and experiences regarding specific elements of the transition process [47, 54]. Failure to take adolescents’ perspectives into account leads to routine problematization of adolescents as inadequately equipped to manage transition into adult clinical care [55], yet little research is focused on understanding perspectives of adolescents having difficulties with transition. In our study for instance, it is not surprising that adolescents with a higher level of preparedness reported satisfaction with preparation process for transition in general. However, notable proportions of adolescents reported being somewhat dissatisfied (11.7%) or very dissatisfied (58.3%) with the preparation process in general. Despite the preparation for transition, most adolescents expressed preference to continue accessing services in pediatric clinics. Although anxiety and reluctance to the transfer into adult clinics is not unusual [28, 37, 39, 51, 54], understanding the underlying reasons why adolescents were dissatisfied with the preparation process will be important in facilitating improvement of the transition preparedness process in the study context.

Fourth, an overarching strategy that contributed to the preparation for transition was having an agreed transition readiness protocol in place, whose distinct elements can be evaluated in depth. In this regards, it is particularly important to evaluate how the transition procedure is understood by adolescents, health providers, parents and guardians, and its uniformity which is implemented across different clinics. Despite the presence of a transition protocol, only a small proportion of adolescents had completed a transfer form (2.7%), been allocated with a Case Manager (24.7%), been counselled about the transition process (29.6%) or visited an adult ART clinic (19.7%) as part of the preparation for transition, suggesting that the transition process was not uniformly implemented. Availability and subsequent implementation of transition protocols is a key feature of transition quality [47]. In addition, variation between sites in terms of process implementation as well as conceptualization; e.g., viewing transition as an event rather than a process—can significantly affect the care quality and outcomes after the transition [26, 47].

Finally, results from this study generally indicate areas where additional support may be required by adolescents living with HIV to improve their disease management, coping mechanisms and outcomes, even if these factors did not necessarily affect transition preparedness independently. This includes support with adherence for the few that found it difficult to remember to take medicines, providing skills for the half that did not have a method of keeping track of their health care appointments, increasing HIV disclosure education for parents whose adolescents did not know what medicines they were taking, ongoing support with social and transport, vocational training, ongoing HIV and ART literacy, and information services for those who did not know the nature of their infection, support to cope with stigma, and ensuring adequate supplies of treatments to prevent unnecessary appointments. Although these are not unique challenges as they have been reported elsewhere [17, 24, 25], they could significantly affect ability of adolescents to stay in school instead of working, manage and keep their appointments, cope with HIV diagnosis in the long term, manage their own disclosure to sexual partners, and ultimately their ability to manage HIV on their own. Further data are useful in providing information regarding the best ways in which adolescents can be supported to meet the above needs comprehensively.

## Limitations of the study

Data reported in this study were collected from a diverse sample of major ART clinics in 13 city and provinces with different socio-demographic and geographical characteristics. However, generalizability of these results may be limited by biases resulting from the use of self-reported measures, recall of events that had taken several months or years before the study. The study focused exclusively on large ART clinics, adolescents who could communicate in Khmer and adolescents who were with a parent or guardian available for consent and ability to transport themselves to the clinic. These criteria may limit the generalizability of the study findings to only adolescents who are more affluent, educated and living in urban areas where there tend to be more resources available. One may speculate that this population is more prepared for the transition into adult care. It is possible that factors influencing preparedness for transition could change over time; however, this cross-sectional study captured a snapshot of these factors. In addition, qualitative data will be useful in contextualizing findings reported in this paper.

Analyses and results reported in this paper was based on scale developed and validated elsewhere [43]. While, generalizable tools to assess transition preparedness are limited, different conclusions may have been reached had a different scale been use such as that used by Wiener et al., which classifies preparedness into four categories: poor, moderate, good and excellent [53]. In their study, Wiener et al. [53] assessed six variables: (1) whether a local health care provider had been identified and, if so, (i) whether the participant had made an appointment with this provider, (ii) who made the appointment, and (iii) how comfortable the participant felt calling the provider for advice or turning to this provider for medical care (8 items); (2) if the participant had medical insurance and whether they (i) would need to pay out of pocket for medications and (ii) have the financial ability to cover these costs (4 items); (3) whether the participant had transportation for medical appointments (1 item); (4) whether a pharmacy had been identified and whether the participant had obtained medications and/or refills through this pharmacy (2 items); (5) knowledge of disease status, medication regimens and dosages (5 items); and (6) if they had or knew of a social worker who would be available for assistance with obtaining support and/or services (1 item). The scale yields one global readiness score, which is used to classify participants’ preparedness as either poor, moderate, good or excellent.

It is notable that the self-reported perception of preparedness was somewhat higher than the assessment using the scale, as 13 and 75.3% of participants reported feeling very prepared and somewhat prepared, respectively on a 4-item Likert scale. These differences may be due to social desirability bias, or genuine shortcomings in how the scale used in this study assessed preparedness, especially given that it dichotomises preparedness into two categories as opposed to the scale used by Wiener et al. referred to above. Nevertheless, the overlap of some factors included in these two scales, together with our own pre-testing of the former scale suggested that the factors we focused on were highly relevant to the study setting. At the same time, transition preparedness could be affected by physiological, psychological or age-related developmental or factors. For instance, Wiener et al. [53] went further to include a separate assessment of anxiety within their study, and found that over time, transition preparedness improved, while the state of anxiety decreased. Although we did not examine the nature of psychological influences on transition preparedness in depth—which could be explored in future research, our study provided valuable information regarding the implementation of the transition protocol and highlighted areas that require attention.

## Conclusions

This study reports individual, social, and health system and services factors that contribute to successful transition preparedness in the context of a clear and structured transition protocol for adolescents living with HIV in Cambodia, a substantial proportion of whom were prepared for the transition to adult services. Nevertheless, gaps were identified, which will require to be addressed to ensure that age-appropriate and individualized case management for transition is enhanced and uniformly implemented across different sites. Transition process and interventions will also need to concurrently address contextual issues facing adolescents living with HIV, by focusing on disclosure, sexuality, stigma, social protection, and social support, as well as HIV education and ART literacy, while at the same time creating supportive family, peer, and healthcare environments for adolescent transition. To achieve these goals, adolescents, parents, and health providers should be involved.

## Abbreviations

AIDS: acquired immune deficiency syndrome
AOR: adjusted odds ratio
CI: confidence interval
HIV: human immunodeficiency virus infection
NECHR: National Ethics Committee for Health Research
NGO: non-governmental organization
SD: standard deviation
WHO: World Health Organization
